# Serum- and xeno-free culture of human umbilical cord perivascular cells for pediatric heart valve tissue engineering

**DOI:** 10.1186/s13287-023-03318-3

**Published:** 2023-04-19

**Authors:** Shouka Parvin Nejad, Monica Lecce, Bahram Mirani, Nataly Machado Siqueira, Zahra Mirzaei, J. Paul Santerre, John E. Davies, Craig A. Simmons

**Affiliations:** 1 Translational Biology and Engineering Program, Ted Rogers Centre for Heart Research, Toronto, Canada; 2 Institute of Biomedical Engineering, University of Toronto, Toronto, Canada; 3 Department of Mechanical and Industrial Engineering, University of Toronto, Toronto, Canada; 4 Faculty of Dentistry, University of Toronto, Toronto, Canada; 5 Tissue Regeneration Therapeutics, Toronto, Canada

**Keywords:** Mesenchymal stromal cells, Human umbilical cord perivascular cells, Serum- and xeno-free culture, Extracellular matrix, Heart valve tissue engineering

## Abstract

**Background:**

Constructs currently used to repair or replace congenitally diseased pediatric heart valves lack a viable cell population capable of functional adaptation in situ, necessitating repeated surgical intervention. Heart valve tissue engineering (HVTE) can address these limitations by producing functional living tissue in vitro that holds the potential for somatic growth and remodelling upon implantation. However, clinical translation of HVTE strategies requires an appropriate source of autologous cells that can be non-invasively harvested from mesenchymal stem cell (MSC)-rich tissues and cultured under serum- and xeno-free conditions. To this end, we evaluated human umbilical cord perivascular cells (hUCPVCs) as a promising cell source for in vitro production of engineered heart valve tissue.

**Methods:**

The proliferative, clonogenic, multilineage differentiation, and extracellular matrix (ECM) synthesis capacities of hUCPVCs were evaluated in a commercial serum- and xeno-free culture medium (StemMACS™) on tissue culture polystyrene and benchmarked to adult bone marrow-derived MSCs (BMMSCs). Additionally, the ECM synthesis potential of hUCPVCs was evaluated when cultured on polycarbonate polyurethane anisotropic electrospun scaffolds, a representative biomaterial for in vitro HVTE.

**Results:**

hUCPVCs had greater proliferative and clonogenic potential than BMMSCs in StemMACS™ (*p* < 0.05), without differentiation to osteogenic and adipogenic phenotypes associated with valve pathology. Furthermore, hUCPVCs cultured with StemMACS™ on tissue culture plastic for 14 days synthesized significantly more total collagen, elastin, and sulphated glycosaminoglycans (*p* < 0.05), the ECM constituents of the native valve, than BMMSCs. Finally, hUCPVCs retained their ECM synthesizing capacity after 14 and 21 days in culture on anisotropic electrospun scaffolds.

**Conclusion:**

Overall, our findings establish an in vitro culture platform that uses hUCPVCs as a readily-available and non-invasively sourced autologous cell population and a commercial serum- and xeno-free culture medium to increase the translational potential of future pediatric HVTE strategies.

**Graphical Abstract:**

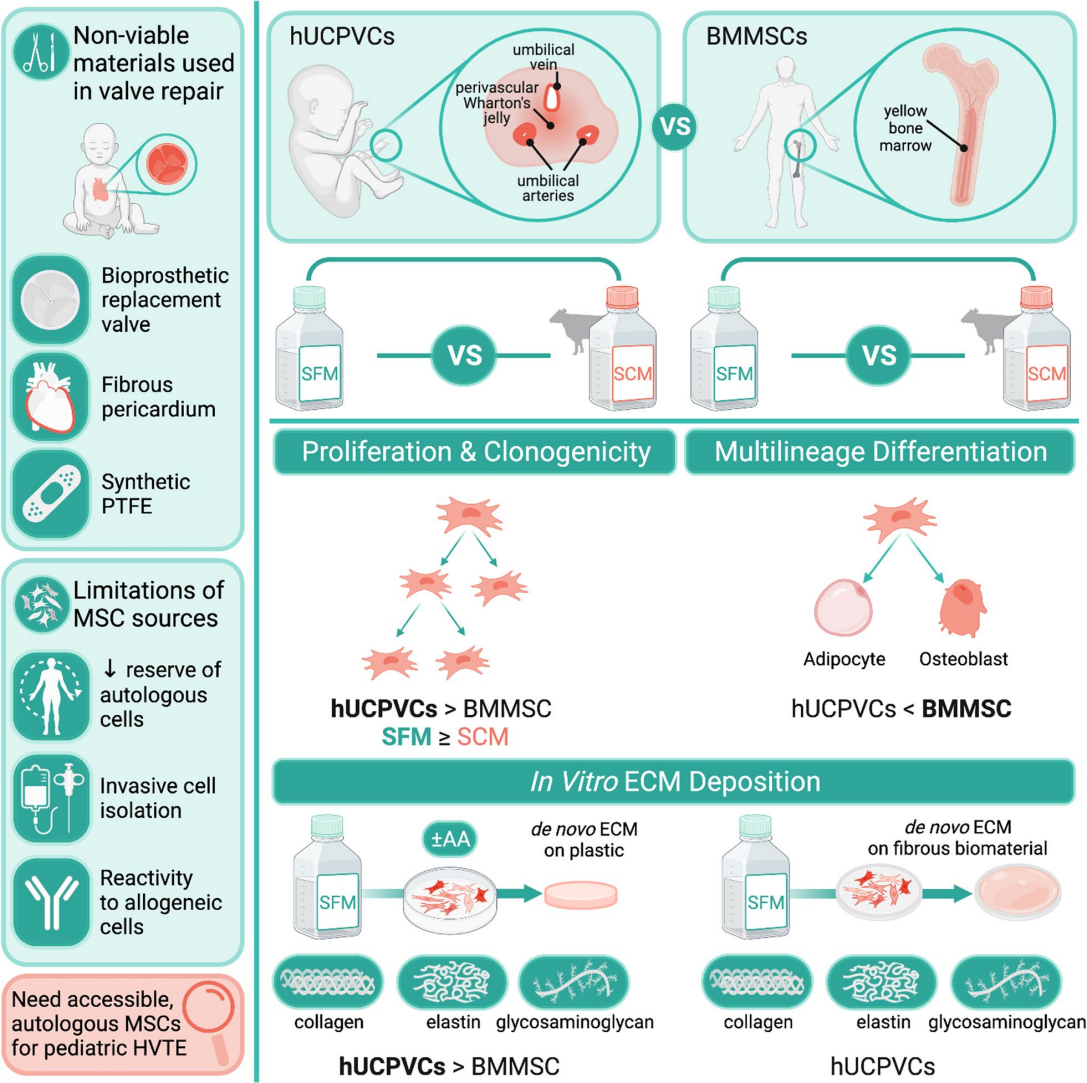
This study evaluated the proliferative, differentiation and extracellular matrix (ECM) synthesis capacities of human umbilical cord perivascular cells (hUCPVCs) when cultured in serum- and xeno-free media (SFM) against conventionally used bone marrow-derived MSCs (BMMSCs) and serum-containing media (SCM). Our findings support the use of hUCPVCs and SFM for in vitro heart valve tissue engineering (HVTE) of autologous pediatric valve tissue. Figure created with BioRender.com.

**Supplementary Information:**

The online version contains supplementary material available at 10.1186/s13287-023-03318-3.

## Introduction

Despite significant advances in interventional cardiology and cardiac surgery [1, 2], valvular repair and replacement procedures still cannot ensure long-term functionality and freedom from reoperation for pediatric patients with congenital heart defects (CHDs), largely because of deficits inherent to valve repair materials and prosthetic replacement valves themselves. In particular, the lack of a viable cell population associated with these interventions renders them incapable of somatic growth, repair, and functional adaptation in response to changing biochemical and biomechanical cues.

Heart valve tissue engineering (HVTE) holds the potential to resolve the currently unmet need for valvular repair and replacement tissue that can grow, self-repair, and remodel to support life-long performance. HVTE strategies can be broadly stratified into in vitro, in situ*,* and in vivo HVTE, with the former two being the most prevalent approaches [3, 4]. In vitro HVTE strategies couple resorbable biomaterials and stromal cells with the aim of producing cellularised living replacement neo-tissue with the compositional, architectural and mechanical properties to function in vivo. While the biomaterial provides a structurally and mechanically robust scaffolding on which extracellular matrix (ECM) is deposited, the presence of a viable cell population in tissue engineered heart valves (TEHVs) ensures functional adaptation and remodelling of the ECM, analogous to the valvular interstitial cell population resident within the native valve [5]. In situ TEHVs encompass bioengineered valve replacements that are decellularized prior to implantation and rely on infiltration of endogenous host cells to populate the construct in vivo. In situ strategies most often use decellularized in vitro TEHVs, and less frequently, acellular bioresorbable scaffolds or decellularized allogeneic or xenogeneic valves. Thus, all in vitro and a majority of in situ HVTE strategies are entirely contingent on the stromal cell source to synthesize structural ECM proteins and assemble tissue in vitro.

HVTE strategies have historically used mesenchymal stromal cells (MSCs) sourced from vascular, dermal, adipose and bone marrow tissues, as they resemble the fibroblast subpopulation of the native valve and can undergo myofibrogenesis to synthesize the three main constituent ECM proteins of the valve leaflet: collagen, elastin, and glycosaminoglycans [6–9]. In the context of pediatric HVTE, MSCs derived from conventional tissue sources are constrained by at least one of three limitations: (i) the invasive nature of cell isolation; (ii) a limited reserve of autologous MSCs; and (iii) immunogenicity associated with allogeneic major histocompatibility complex I-mismatched MSCs [10, 11]. Thus, fetal MSCs derived from prenatal (amniotic fluid, amniotic membrane) and early postnatal (placenta, umbilical cord blood, umbilical cord matrix) tissues have been studied for their utility to pediatric HVTE [12–14].

Among fetal sources of MSCs, the umbilical cord is a promising candidate, as a rich source of autologous progenitor cells that can be harvested non-invasively from tissue that is typically discarded. Human umbilical cord perivascular cells (hUCPVCs) harvested from the perivascular region of the Wharton’s jelly (WJ) possess characteristic properties of MSCs [15–17] in accordance with the minimal criteria set by the International Society for Cell and Gene Therapy (ISCT) MSC committee [18] - hUCPVCs are plastic adherent, differentiate to osteogenic, adipogenic and chondrogenic lineages, and present appropriate cell surface markers (CD105+, CD73+, CD90+, CD45−, CD34−, CD14−, HLA-DR−) [16, 19–21]. Additionally, hUCPVCs contain a high frequency of colony forming unit-fibroblasts (CFU-F) that maintain proliferative and multilineage differentiation potential [22]. Their proven regenerative capacity in dermal wound healing [23], musculoskeletal repair [22], and acute myocardial infarction [16] make a compelling case for their use as autologous progenitor cells for pediatric HVTE. While in vitro cardiovascular tissue engineering strategies have taken advantage of umbilical cord-derived MSCs to synthesize neo-tissue [24–30], these findings cannot be projected to hUCPVCs due to a lacking consensus in the literature with respect to both the anatomical descriptors of the specific regions from which umbilical cord MSCs were harvested and the methodological techniques used to harvest these cells. Furthermore, previous studies of umbilical cord-derived MSCs for in vitro cardiovascular tissue engineering have been conducted in xenogenic serum-supplemented culture conditions, complicating their translational potential.

Clinical translation of TEHVs produced from the combination of biomaterial scaffolds and hUCPVCs ultimately necessitates reproducible in vitro culture conditions. However, supplementation of cell culture medium with undefined xenogenic serum poses a challenge to good manufacturing practices (GMP) as (i) xenogenic serum-containing media (SCM) carries an inherent risk of viral, bacterial, or prion disease transmission from the donor to culture adapted human MSCs [31, 32]; (ii) the presence of xenogenic proteins in engineered constructs cultured with serum can provoke an immune response in a human recipient, undermining the use of autologous cells [33]; and (iii) the undefined composition of serum and lot-to-lot variability resulting from differences in animal husbandry can induce variable responses in culture adapted cells [34, 35].

Here we report serum- and xeno-free culture of hUCPVCs for in vitro HVTE applications and benchmark them against human bone marrow-derived MSCs (BMMSCs), the most pervasively used MSCs in regenerative applications. We show that a commercially available serum- and xeno-free culture medium supports the proliferative capacity of hUCPVCs and BMMSCs and establish serum- and xeno-free in vitro culture conditions to promote deposition of the ECM proteins critical to the structure and function of valve tissue (collagen, elastin, and glycosaminoglycans). Finally, we confirm ECM synthesis by hUCPVCs on electrospun scaffold sheets in a preliminary study of engineered PV repair constructs. This study establishes hUCPVCs as a suitable cell source for HVTE, supported by their accessibility, rapid proliferation, and capacity to generate neo-tissue under serum- and xeno-free culture conditions.

## Materials and Methods

### Cell sources and expansion

Adult human BMMSCs from three male donors were used in this study. BMMSCs from one donor were obtained at passage 1 from Texas A&M Health Science Centre College of Medicine Institute for Regenerative Medicine at Scott & White through a grant from ORIP of the NIH, grant #P40OD011050 (Donor #8013L). Cells were subsequently expanded to passage 5 in SCM consisting of $a$-MEM (Gibco™, cat #12483-020) supplemented with 2–4 mM L-glutamine (Sigma-Aldrich, cat #G7513), 16.7% fetal bovine serum (Gibco™, cat #12561-056), and 1% penicillin/streptomycin (Life Technologies, cat #15140122). BMMSCs from Donor #8013L were used at passage 5 for all experiments. BMMSCs from the remaining two donors were obtained at passage 1 from Lonza (cat #PT-2501, Donor #36461 and Donor #36550) and expanded to passage 3 using MSCGM™ Stem Cell Growth Media Bulletkit (Lonza, cat #PT-3001) as per the vendor’s recommendations. BMMSCs from Donor #36461 and Donor #36550 were used at passage 3 for all experiments. hUCPVCs from full-term umbilical cords of three male neonatal donors (Donor #0917003; Donor #0917004; and Donor #0317004) were isolated and expanded by Tissue Regeneration Therapeutics Inc. (Toronto, Ontario, Canada) using a proprietary protocol and provided in-kind at passage 1. hUCPVCs were expanded to passage 3 using StemMACS™ MSC Expansion Media Kit XF (henceforth referred to as StemMACS™) (Miltenyi Biotec, cat #130-104-182) supplemented with 5 mL of a concentrated antibiotic cocktail consisting of 0.6 mg amphotericin-B (Sigma cat #A9528), 200 mg penicillin (Bioshop Canada, cat #PEN333.25), 100 mg gentamicin sulfate (Sigma, cat #G1397) in 20 mL of phosphate buffered saline (PBS). hUCPVCs from all three donors were used at passage 3 for all experiments.

### Proliferation in StemMACS™

hUCPVCs and BMMSCs from each donor were seeded in 6-well tissue culture plates in triplicate at a density of 3000 cells/cm^2^ and cultured in either StemMACS™ or SCM. Cell culture media was refreshed every three days. Cells were cultured to specific time-points (2, 4, 6, and 8 days in culture) before dissociation by TrypLE (Gibco™, cat # 12604021) and 125 CDU/mL collagenase (Sigma, cat #C0130). Harvested cells were subsequently stained with trypan blue and viable cells were manually counted using a hemocytometer. Average population doubling time over 6 days (day 2–8) was calculated using the following equation: $\mathrm{Doubling \,Time} (\mathrm{hrs})= t*(\mathrm{log}(2))/\mathrm{log}\left(\frac{{N}_{t}}{{N}_{0}}\right)$, where* N*_0_ is the average number of live cells harvested from one well of a 6-well plate on day 2 and* N*_t_ is the number of live cells after 8 days of culture.

### CFU-F frequency in StemMACS™

hUCPVCs and BMMSCs from each donor were cultured in T25 culture flasks in either StemMACS™ or SCM. Once cells reached 75–80% confluence (assessed visually) they were detached from culture flasks by TrypLE, resuspended in either StemMACS™ or SCM, and subsequently plated at a density of 100 cells/well in 6-well tissue culture plates in triplicate. On day 7, samples were washed with PBS, stained with 2.5% crystal violet in 100% ethanol for 1 h at room temperature, and rinsed with distilled water. Stained colonies were first visually identified in the wells and counted as a colony upon confirmation of 8 or more cells within the cluster using brightfield microscopy.

### Osteogenic and adipogenic differentiation potential

Osteogenic and adipogenic differentiation potential were assessed in accordance with the ISCT minimal criteria for MSCs [18]. hUCPVCs and BMMSCs from each donor were seeded in 6-well tissue culture plates at a density of 3000 cells/cm^2^ with SCM. At 75–80% confluence (assessed visually), cell culture media was replaced with either osteogenic induction media, adipogenic induction media, or control SCM. Each cell donor was cultured in triplicate under each media condition. Osteogenic differentiation medium consisted of SCM supplemented 10 nM dexamethasone (Sigma, cat #D2915), 20 μM $\beta$-glycerolphosphate (Sigma, cat #G9891), and 50 μM L-ascorbic acid 2-phosphate (Sigma, cat #A8960). Adipogenic differentiation medium consisted of SCM supplemented with 0.5 μM dexamethasone (Sigma, cat #D2915), 0.5 μM isobutylmethylxanthine (Sigma, cat #I5879), and 50 μM indomethacin (Sigma, cat #I7378). Cells were cultured for up to 20 days after induction, and media was refreshed every 4 days. Due to cell peeling and rolling, hUCPVCs from one biological donor (Donor #0917004) were fixed on day 15 of induction with differentiation medium. Cells were fixed with 10% neutral buffered formalin (NBF) (Sigma, cat #HT501128) and stored in PBS at 4 °C prior to staining. Osteogenic and adipogenic differentiation was assessed by staining with 2% alizarin red solution (Electron Microscopy Sciences, cat #2620601) and 0.3% (w/v) oil red-O (Sigma, cat #00625) solution respectively, using a protocol provided by Texas A&M Health Science Centre College of Medicine Institute for Regenerative Medicine.

### ECM protein synthesis

hUCPVCs and BMMSCs from each donor were seeded in 12-well tissue culture plates with StemMACS™ cell culture media at a density of 3000 cells/cm^2^. Cell culture media was changed on day 1 and replaced with either StemMACS™ or 50 µM ascorbic acid (AA) (Sigma, cat #A4544) supplemented StemMACS™. Cell culture media was refreshed every 1–2 days. AA-supplemented culture media was prepared fresh at each media change. Cells were cultured for 14 days. Due to observations of cell peeling and rolling among the BMMSC donors, samples were harvested after 9 (Donor #36461 and Donor #36550) and 11 (Donor #8013L) days in culture. Samples were harvested by either papain [36] or oxalic acid digestion. Detailed methodology for papain and oxalic acid digestions are provided in Additional file 1.

Total DNA, sulfated glycosaminoglycan (s-GAG), and hydroxyproline content of papain digested lysates was quantified using the Hoechst dye 33258 assay [37], dimethylmethylene blue dye binding assay [38], and chloramine-T/Ehrlich’s reagent assay [39], respectively. The insoluble elastin content of oxalic acid digested samples was measured using the Fastin™ Elastin Assay Kit by Biocolor (Accurate Chemical and Scientific Corporation, cat. #CLRF2000). Additional details on biochemical assays are provided in Additional file 1.

### Fabrication and characterization of electrospun PCNU constructs

Polycarbonate polyurethane (PCNU) was synthesized as previously described [40]. Briefly, the base PCNU was synthesized from the reaction of poly(1,6-hexyl 1,2-ethyl carbonate)diol, 1,6-hexane diisocyanate, and 1,4-butanediol in N,N-dimethylacetamide at a molar ratio of 3:2:1, under an inert atmosphere of nitrogen. The polystyrene equivalent weight average molecular weight was estimated between 99 × 10^3^ and 105 × 10^3^ g/mol by gel permeation chromatography. Anionic dihydroxyl oligomer (ADO) was added to PCNU at a concentration of 0.15% (w/w) and dissolved in 1,1,1,3,3,3-hexafluoro-2-propanol to produce an 18% (w/v) PCNU polymer solution. The solution was electrospun through an 18-guage stainless steel needle at a flow rate of 0.5 mL/hr onto a rotating mandrel (1150 rpm), at a distance of 18 cm, and an applied voltage of 18 kV (+ 17 kV at the needle and − 1 kV at mandrel) into nanofibrous PCNU scaffolds. Scaffolds were dried overnight in a vacuum oven at 45 °C.

To confine hUCPVCs to electrospun PCNU scaffolds for cell culture, circular discs were removed from one electrospun sheet using a biopsy punch. The PCNU discs were placed over the opening of a 200 μL microfuge tube which had been modified to form a culture vessel by cutting off the end of the tube and excising the inner aspect of the tube cap, as previously described [40, 41]. The scaffold was held in place between the rim of the cap and the inner wall of the microfuge tube. PCNU discs in culture vessels were placed in 24-well plates (1 construct/well) and sterilized with ethylene oxide. Due to spatial heterogeneity within one sheet, each isolated region was counted as an independent sample.

Cell-free sterilized PCNU discs were isolated from culture vessels for characterization by scanning electron microscopy (*N* = 3) and biaxial mechanical testing (*N* = 3). Scanning electron microscopy was used to visualize scaffold fibres. Scanning electron micrographs were imported into Image J software for analysis of fibre diameter and alignment using the Diameter J plugin. Square samples (4.5 mm × 4.5 mm) were isolated from PCNU discs using a specimen cutter and underwent displacement controlled equibiaxial force testing (Biotester 5000; Cellscale, Waterloo, CAN) in a PBS bath at 37 °C. Samples were mounted to the mechanical system using a tine attachment system (Biorakes with 0.7 mm tine spacing, CellScale, Waterloo, CAN), and a biaxial tensile testing protocol previously described by Labrosse et al. [42] was used. Strain tracking was conducted in the Biotester 5000’s LabJoy software, and a MATLAB code from Labrosse et al. [42] was used to generate Green strain and membrane tension outputs.

### hUCPVC culture on electrospun PCNU scaffolds in StemMACS™

Sterilized PCNU scaffolds in modified culture vessels were pre-coated with 50 μL 1 mg/mL human plasma derived fibronectin (Sigma, cat #FC010) at a coating density of 2.5 μg/cm^2^ in PBS overnight at 4 °C. Fibronectin solution was subsequently aspirated and scaffolds were washed with pre-warmed PBS (with calcium and magnesium) (37 °C) prior to cell seeding. hUCPVCs from one donor (Donor #0917003) were seeded on PCNU scaffolds at a density of 50,000 cells/cm^2^ with 50 µL StemMACS™ culture media in modified culture vessels. An additional 500 μL/well of culture media was added to the 24-well plates housing the modified culture vessels. Cell culture media was replaced every other day.

### Cell morphology on electrospun PCNU scaffolds in StemMACS™

After 14 and 21 days in culture, hUCPVC-seeded PCNU constructs (*N* = 3 per timepoint) were harvested for assessment of cell morphology by staining with FITC-labelled phalloidin (Sigma, cat #P5282) F-actin and Hoechst 33342 (Sigma, cat #B2261) nuclear stains before imaging by confocal microscopy. Details of the staining protocol are provided in Additional file 1.

Confocal images collected from three different locations on each construct were imported into Image J software for analysis of F-actin alignment. Z-stacks from the three locations on each sample were stratified into two groups to illustrate hUCPVCs in direct contact with PCNU scaffold fibres (PCNU-contacting) and those that had grown on top of the PCNU-contacting cells (cell-contacting). F-actin fibre alignment was quantified using the Orientation J vector field plugin in Image J.

### ECM protein synthesis on electrospun PCNU scaffolds in StemMACS™

After 14 and 21 days in culture, hUCPVC-seeded PCNU constructs (*N* = 3 per timepoint) were harvested for digestion by papain (day 14 and 21) and oxalic acid (day 21). Cell-free PCNU constructs that were incubated in parallel with cell-seeded scaffolds were similarly digested to serve as negative controls for subsequent analyses. DNA, hydroxyproline, s-GAG and $\alpha$-elastin content associated with the cell-seeded constructs was quantified using the biochemical assays described above. Detailed methodology of digestion protocols and biochemical assays are provided in Additional file 1.

To visualize ECM proteins, hUCPVC-seeded PCNU constructs were stained using Movat’s pentachrome. Briefly, samples were harvested on day 21 of culture, embedded in optimal cutting temperature (OCT) compound and snap-frozen using liquid nitrogen. OCT-embedded samples were cryosectioned (10 µm) and subsequently stained with Movat’s pentachrome. Stained sections were imaged on a brightfield microscope.

### Statistical analysis

Graphical visualization of data and statistical analysis were performed using GraphPad Prism 9. Statistical differences between conditions were assessed using either two-way analysis of variance (ANOVA) with Tukey’s multiple comparisons test or Student's t-test. All experimental data are presented as mean ± standard error of the mean (SEM).

## Results

### Proliferation in StemMACS™

**Fig. 1:**
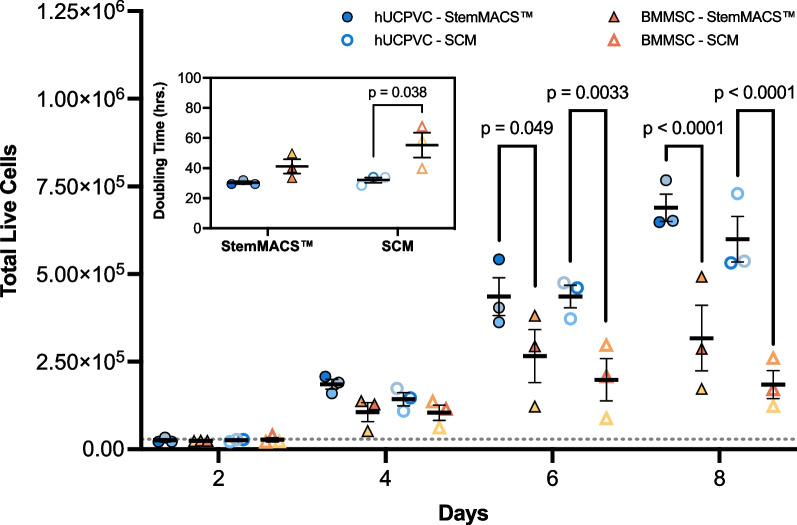
Proliferation and population doubling time of hUCPVCs (*N* = 3 donors, *n* = 3 technical replicates per donor) and BMMSCs (*N* = 3 donors, *n* = 3 technical replicates per donor) in xeno-free StemMACS™ and SCM. StemMACS™ culture medium supported the proliferation of both hUCPVCs and BMMSCs similarly to conventionally used SCM in an 8-day proliferation assay. Proliferation of hUCPVCs in each media formulation was similar to that of BMMSCs after 2 and 4 days in culture. After 6 and 8 days in culture, there were significantly more hUCPVCs than BMMSCs in both StemMACS™ (Day 6: *p* = 0.049; Day 8: *p* < 0.0001) and SCM (Day 6: *p* = 0.0033; Day 8: *p* < 0.0001) media conditions, suggesting a superior proliferative capacity of hUCPVCs. *Inset:* The average population doubling time of hUCPVCs was lower than that of their BMMSC counterparts in both StemMACS™ and SCM (*p* = 0.038), though this difference was only statistically significant in the latter media condition. Reported values are mean ± SEM; statistical analysis by two-way ANOVA with post-hoc Tukey’s multiple comparisons test. Dashed grey line indicates initial cell seeding density in a 6-well plate. Each shade corresponds to a different donor of hUCPVCs (Donor 0917003; Donor 0917004; Donor 0317004 from dark to light shade of blue) and BMMSCs (Donor 8013L; Donor 36341; Donor 36550 from light to dark shade of yellow/orange)

Proliferation of hUCPVCs and BMMSCs was compared in StemMACS™ culture medium and SCM. For both cell types, proliferation was similar in StemMACS™ versus SCM (Fig. 1). However, hUCPVCs proliferated more rapidly than BMMSCs in both StemMACS™ and SCM, with significantly more cells at day 6 (*p* < 0.05) and day 8 (*p* < 0.0001) (Fig. 1). Accordingly, the average population doubling time of hUCPVCs was shorter than that of BMMSCs in both StemMACS™ and SCM conditions, although this difference was only statistically significant in the SCM cohort (*p* = 0.038) (Fig. 1, inset).

### Frequency of CFU-F in StemMACS™

**Fig. 2:**
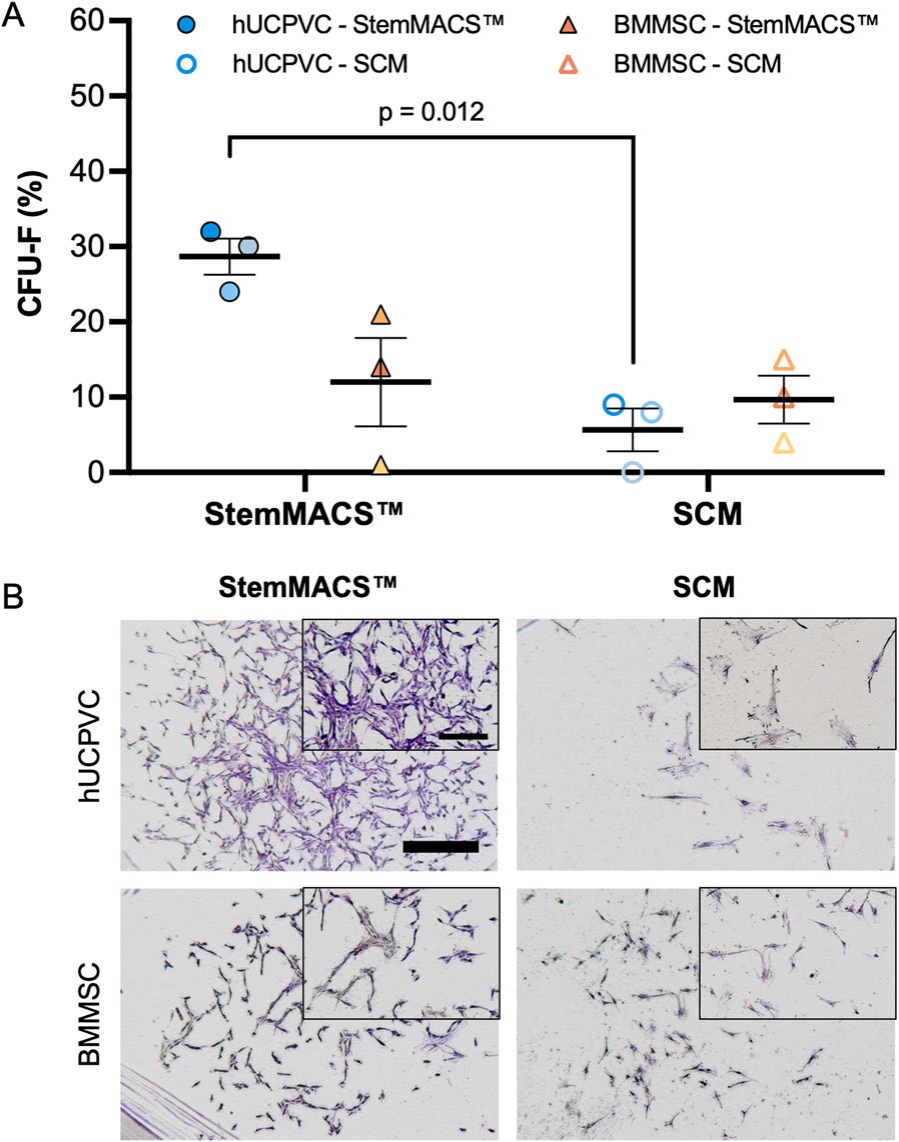
**A** Frequency of CFU-F in hUCPVCs and BMMSCs (*N* = 3 donors each, *n* = 3 technical replicates per donor) cultured in StemMACS™ and SCM. Culture with StemMACS™ resulted in a similar frequency of CFU-F compared to culture with SCM for BMMSCs. hUCPVCs cultured in StemMACS™ had a significantly higher frequency of CFU-F than their counterparts cultured in SCM (*p* = 0.012). Reported values are mean ± SEM; statistical analysis by two-way ANOVA with post-hoc Tukey’s multiple comparisons test. Each shade corresponds to a different donor of hUCPVCs (Donor 0917003; Donor 0917004; Donor 0317004 from dark to light shade of blue) and BMMSCs (Donor 8013L; Donor 36341; Donor 36550 from light to dark shade of yellow/orange). **B** Crystal violet stained CFU-F colonies imaged under brightfield microscopy (scale bar = 1000 μm; inset scale bar = 500 μm). hUCPVCs cultured in StemMACS™ produced larger and more densely populated colonies than those cultured in SCM. BMMSCs consistently had smaller and less densely populated colonies than hUCPVCs in both media conditions

The frequency of CFU-F in BMMSCs and hUCPVCs was assessed in StemMACS™ and SCM. BMMSCs had similar CFU-F frequencies (Fig. 2A) and colony size and density (Fig. 2B) in in StemMACS™ medium and SCM. In contrast, hUCPVCs cultured in StemMACS™ had a significantly higher frequency of CFU-F than those in SCM (*p* = 0.012) (Fig. 2A). Furthermore, hUCPVCs in StemMACS™ had notably larger and more densely populated colonies than their SCM cultured counterparts (Fig. 2B). No other conditions were statistically different, indicating that serum- and xeno-free StemMACS™ supported the clonal expansion of BMMSCs as well as SCM, while enhancing the clonal expansion of hUCPVCs compared to SCM.

### Osteogenic and adipogenic differentiation potential

**Fig. 3:**
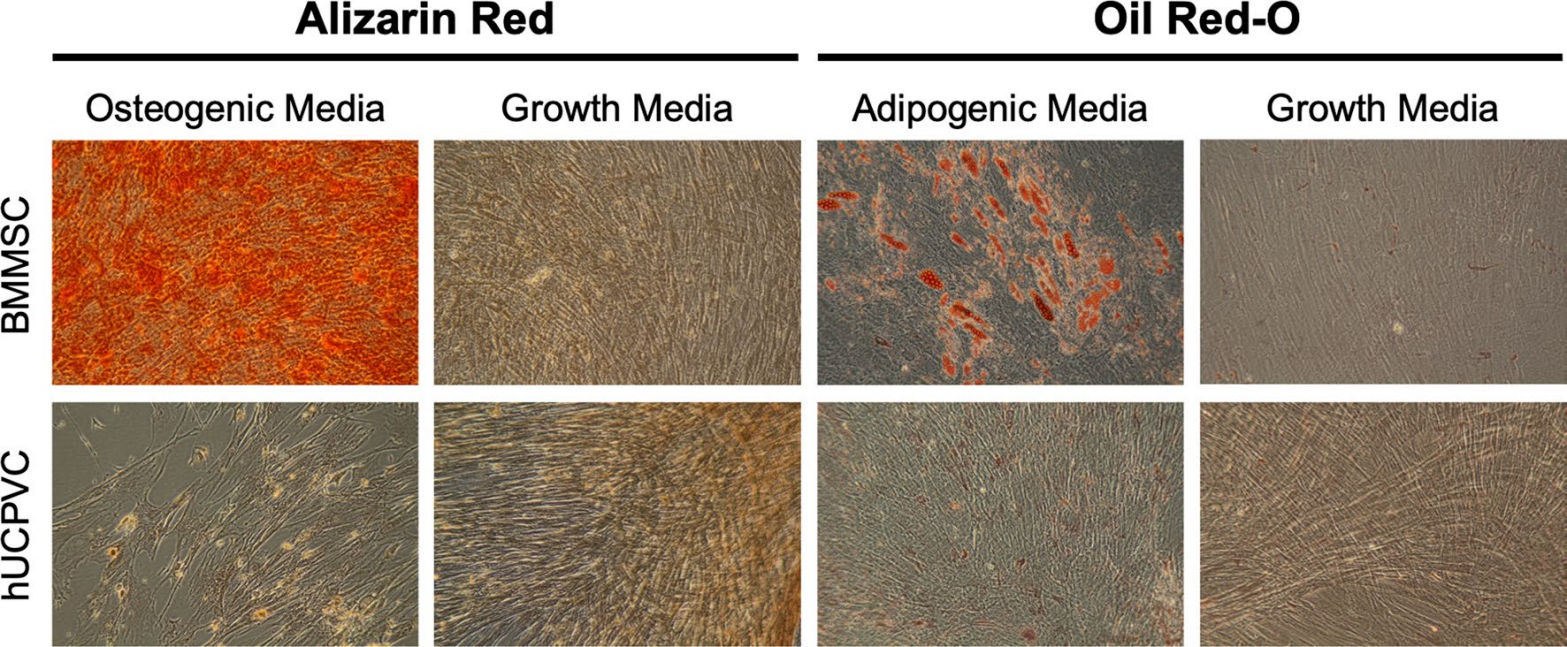
BMMSCs from all donors cultured in either osteogenic or adipogenic culture medium differentiated to osteogenic and adipogenic lineages as indicated by positive alizarin red and oil red-O staining, respectively. Conversely, none of the hUCPVC donors differentiated to either lineage. hUCPVC and BMMSC negative controls cultured in control SCM did not show differentiation to osteogenic or adipogenic lineages. Representative image from one biological donor shown for each cell type

All donors of BMMSCs demonstrated osteogenic differentiation potential as evidenced by positive alizarin red staining indicating calcium deposits (Fig. 3). Similarly, BMMSCs cultured in adipogenic media showed a capacity for adipogenesis as shown by positive oil red-O staining of lipid droplets (Fig. 3). When cultured in growth medium, BMMSCs did not differentiate to osteogenic or adipogenic lineages. hUCPVCs cultured in osteogenic induction media did not show evidence of differentiation after staining with alizarin red, similar to their growth medium cultured controls. Evidence of adipogenesis was also absent from hUCPVCs cultured in adipogenic culture medium and their growth medium cultured controls. Overall, after induction with the appropriate culture media, hUCPVCs did not demonstrate differentiation potential to either osteogenic or adipogenic lineages.

### ECM synthesis on tissue culture polystyrene in StemMACS™

Having established that StemMACS™ culture medium supported the proliferative capacity and CFU-F frequency of hUCPVCs and BMMSCs, we then investigated in vitro ECM synthesis by each cell type in serum- and xeno-free culture conditions. hUCPVC and BMMSCs were cultured in either StemMACS™ medium alone or StemMACS™ supplemented with 50 µM AA for up to 14 days on tissue culture polystyrene (TCPS).

**Fig. 4:**
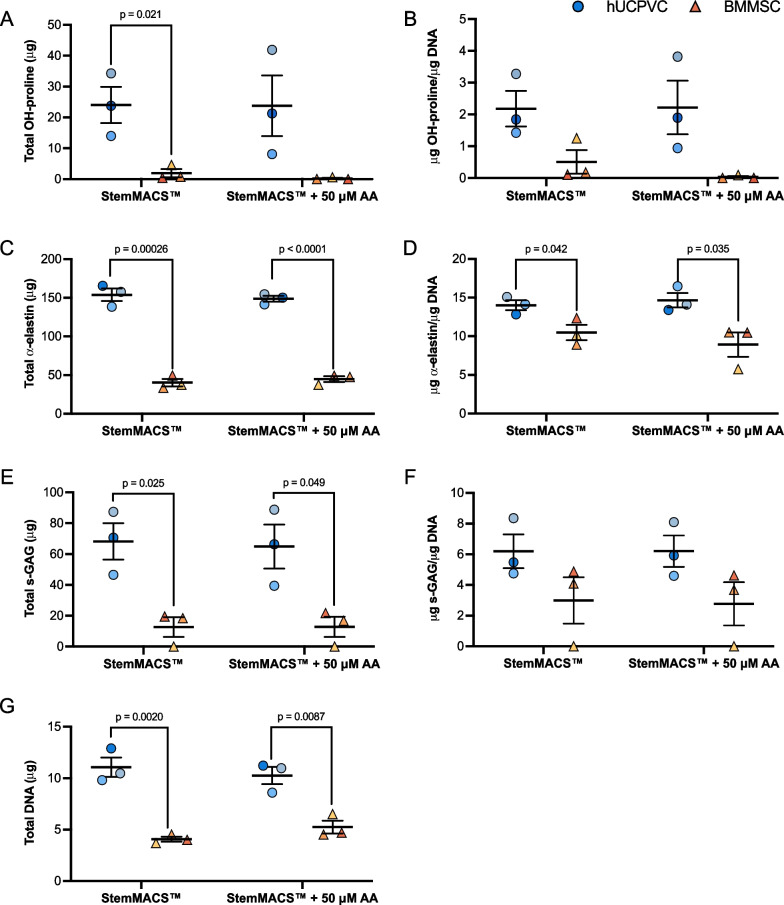
Quantification of total and DNA-normalized hydroxyproline (OH-proline) (**A**, **B**), elastin (**C**, **D**), and sulphated glycosaminoglycans (s-GAG) (**E**, **F**) synthesized by hUCPVCs and BMMSCs (*N* = 3 donors each, *n* = 3 technical replicates per donor) cultured in StemMACS™ and ascorbic acid (AA)-supplemented StemMACS™ culture media. hUCPVCs synthesized significantly more total **A** OH-proline (*p* = 0.021), **C** elastin (*p* = 0.00026) and **E** s-GAG (*p* = 0.025) than their BMMSC counterparts cultured in StemMACS™. Supplementation with 50 μM AA did not significantly alter ECM protein synthesis in either cell population. Similarly, DNA-normalized **B** OH-proline, **D** elastin, and **F** s-GAG content in hUCPVC cultures was higher than that of BMMSCs in both StemMACS™ and AA-supplemented StemMACS™ culture media, although this difference was only statistically significant for DNA-normalized elastin content (*p* = 0.042 and *p* = 0.035, respectively). **G** Total DNA content of hUCPVCs cultured in StemMACS™ and AA-supplemented StemMACS™ culture media was significantly greater than that of their BMMSC counterparts (*p* = 0.0020 and *p* = 0.0087). Reported values are mean ± SEM; statistical analysis by unpaired t-test. Each shade corresponds to a different donor of hUCPVCs (Donor 0917003; Donor 0917004; Donor 0317004 from dark to light shade of blue) and BMMSCs (Donor 8013L; Donor 36341; Donor 36550 from light to dark shade of yellow/orange)

Total and DNA-normalized hydroxyproline (Fig. 4A, B), $a$-elastin (Fig. 4C, D), and s-GAG content (Fig. 4E, F) were quantified in hUCPVC and BMMSC lysates via biochemical analysis. hUCPVCs synthesized more total hydroxyproline than BMMSCs in StemMACS™ with or without AA supplementation, although this difference was only found to be statistically significant in unsupplemented StemMACS™ (*p* = 0.021) (Fig. 4A). hUCPVCs also synthesized more total $a$-elastin than BMMSCs, in both unsupplemented (*p* = 0.00026) and AA-supplemented conditions (*p* < 0.0001) (Fig. 4C). Finally, total s-GAG synthesized by hUCPVCs was significantly greater than that synthesized by BMMSCs in both StemMACS™ (*p* = 0.025) and AA-supplemented StemMACS™ (*p* = 0.049) (Fig. 4E). Total DNA quantified in hUCPVC lysates was significantly greater than in BMMSCs in both StemMACS™ (*p* = 0.0020) and AA-supplemented StemMACS™ (*p* = 0.0087) (Fig. 4G).

When normalized to total DNA content, hUCPVCs deposited significantly more $a$-elastin per unit DNA than BMMSCs with (*p* = 0.042) and without (*p* = 0.035) AA-supplementation (Fig. 4D). The DNA-normalized hydroxyproline (Fig. 4B) and s-GAG (Fig. 4F) content of hUCPVCs, though greater than that of their BMMSC counterparts, were not significantly different in either basal or AA-supplemented StemMACS™.

### Culturing hUCPVCs on PCNU scaffolds in StemMACS™—Cell morphology and ECM synthesis

**Fig. 5:**
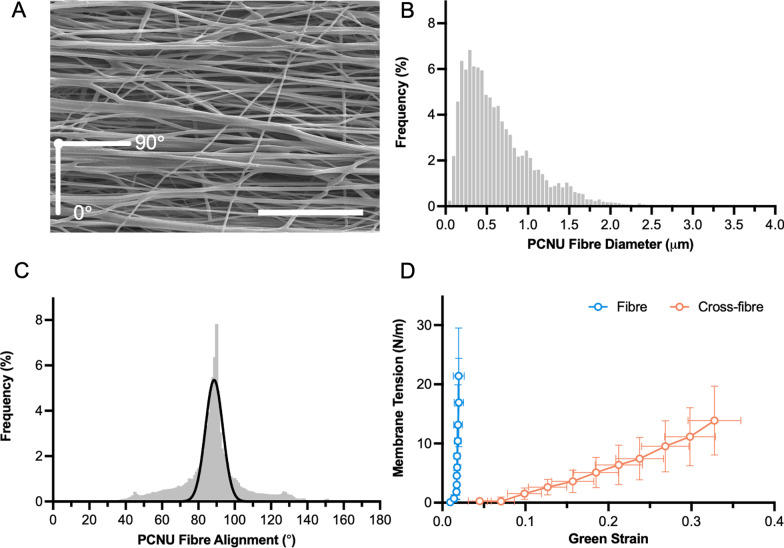
Characterization of PCNU scaffolds by scanning electron microscopy and biaxial tensile testing. **A** Representative scanning electron microscopy image of PCNU fibres in an electrospun biomaterial sheet (scale bar = 20 μm). Three separate regions of the electrospun sheet were isolated for scanning electron microscopy imaging and subsequent quantification of fibre diameter and orientation, and biaxial mechanical properties (*N* = 3). **B** Electrospun PCNU fibres were 447 ± 56.7 nm (mean ± SD) in diameter and **C** followed a principal direction of alignment, that conferred **D** anisotropic tensile properties to the PCNU scaffolds with greater compliance in the cross-fibre direction than the fibre direction

**Fig. 6:**
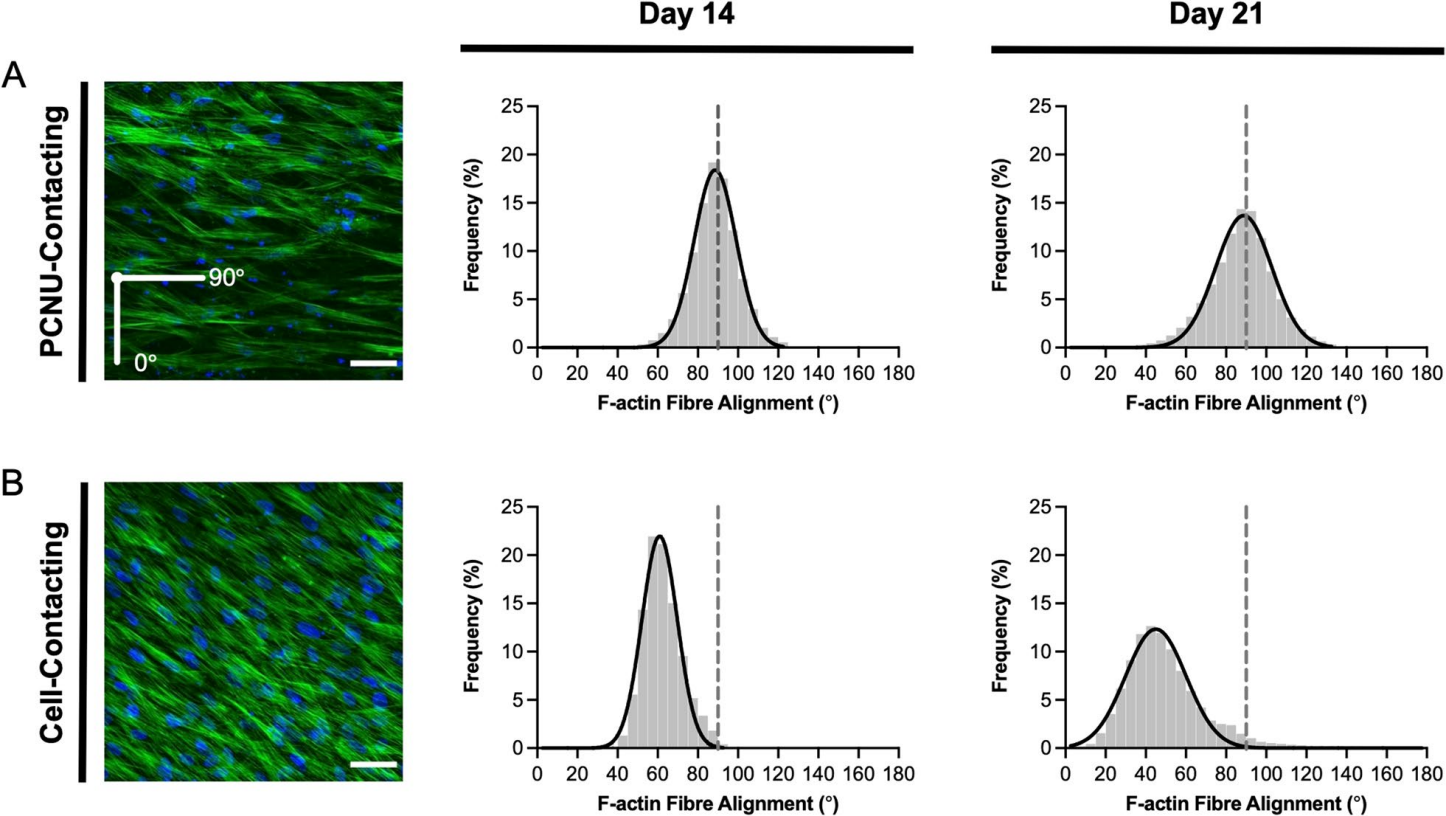
F-actin (green) and nuclear (blue) staining of hUCPVCs cultured on electrospun PCNU for 14 and 21 days in StemMACS™. Confocal images and frequency distribution plot of F-actin alignment in hUCPVC-seeded PCNU scaffolds (*N* = 3) showed two principal angles of cell alignment: **A** hUCPVCs in direct contact with PCNU fibres aligned with the scaffold fibres (90°), whereas **B** hUCPVCs growing on a monolayer of cells away from the surface of the scaffold aligned away from the principal direction of the PCNU fibres. PCNU fibres oriented at 90° marked by a grey dashed line on frequency distribution plots. Confocal images of hUCPVC-seeded PCNU constructs are representative of *N* = 3 on day 14 (scale bar = 50 μm)

Provided with evidence of the superior proliferative and ECM-synthesizing capacity of hUCPVCs over BMMSCs in StemMACS™ culture medium on TCPS, we then evaluated hUCPVCs as a prospective cell source for in vitro HVTE applications when cultured on a suitable biomaterial. hUCPVC were grown on electrospun nanofibrous PCNU scaffolds, a biodegradable biomaterial applicable to HVTE applications due to its highly aligned fibres and anisotropic mechanical behaviour (Fig. 5). After 14 and 21 days in culture, hUCPVC morphology on PCNU scaffolds was assessed by staining cytoskeletal F-actin. Confocal imaging revealed that hUCPVCs followed two principal directions of alignment based on proximity to the PCNU fibres. Cells in direct contact with scaffold fibres showed a high degree of alignment with PCNU fibres (90°) on days 14 (88.62° ± 10.47°) and 21 (88.79° ± 14.02°) (Fig. 6). Moving away from the surface of the PCNU scaffolds, where cells were layered on top of other cells and not in direct contact with the fibres, the direction of hUCPVC alignment shifted away from that of the PCNU fibres (Fig. 6).

**Fig. 7:**
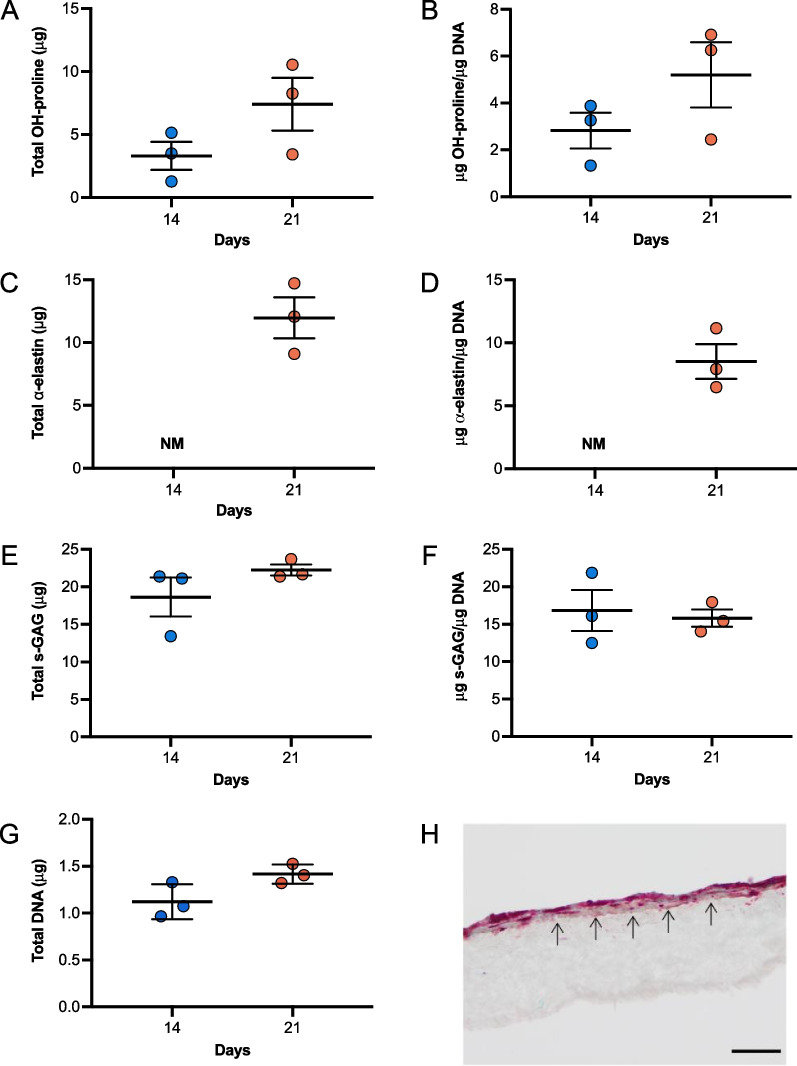
Quantification of total and DNA-normalized hydroxyproline (OH-proline) (**A**–**B**), elastin (**C**–**D**), and sulphated glycosaminoglycans (s-GAG) (**E**–**F**) synthesized by hUCPVCs from a single donor cultured on electrospun PCNU (*N* = 3) for 14 and 21 days in StemMACS™ culture medium. Total **A** OH-proline, **E** s-GAG and **G** DNA content increased between 14 and 21 days in culture, although this change was not statistically significant. Elastin deposition after 14 days of culture was not measured (NM) and time-dependent changes in elastin content were not evaluated. DNA-normalized **B** OH-proline and **D** elastin content was similar to or approaching that on tissue culture polystyrene (Fig. 4B and D), while DNA-normalized s-GAG content was greater than in tissue culture polystyrene (Fig. 4F). **H** Movat’s pentachrome staining of hUCPVC-seeded PCNU constructs shows that the cells and deposited ECM grew in layers above the PCNU scaffold. Black arrows label boundary between hUCPVC and scaffold (scale bar = 50 μm). Reported values are mean ± SEM; statistical analysis by unpaired t-test

Quantification of ECM deposition after 14 and 21 days of culture on PCNU scaffolds revealed that hUCPVCs retained their capacity to synthesize hydroxyproline (Fig. 7A, B), $\alpha$-elastin (Fig. 7C, D) and s-GAG (Fig. 7E, F). When normalized to DNA amount, the hydroxyproline content of hUCPVC-seeded constructs was similar to that on TCPS (Fig. 7B vs Fig. 4B), while DNA-normalized $\alpha$-elastin content was approaching the levels synthesized on TCPS (Fig. 7D vs Fig. 4D). By contrast, DNA-normalized s-GAG content was approximately 2.5 fold greater on PCNU than TCPS (Fig. 7F vs Fig. 4F). Finally, evaluation of the spatial distribution of hUCPVCs cultured on PCNU scaffolds by Movat’s pentachrome staining revealed multiple cell and tissue layers growing atop the PCNU scaffold (Fig. 7F).

## Discussion

Despite their convenience to autologous HVTE for pediatric CHDs [24, 25, 27–30], umbilical cord-derived MSCs are understudied in this context. In particular, hUCPVCs derived explicitly from the cell and ECM rich perivascular sub-region of the cord’s WJ have not been exploited for their utility to pediatric HVTE applications. This knowledge gap is widened by the absence of literature evaluating ECM synthesis under serum- and xeno-free conditions by hUCPVCs for the purpose of HVTE. Here we demonstrated for the first time that hUCPVCs can be successfully grown in serum- and xeno-free culture media to synthesize the constituent ECM proteins of the native valve in vitro, incentivizing their use for the clinical realization of autologous pediatric TEHVs.

Clinical translation of in vitro TEHVs will ultimately require adherence to GMP guidelines to yield a reproducible construct. The inherent lot-to-lot variability and undefined composition of FBS motivate early adoption of serum- and xeno-free culture of candidate cells in regenerative studies as an important step in the realization of protocols that align with GMP guidelines. The first objective of our study was to determine whether a commercially available serum- and xeno-free culture medium, StemMACS™, was comparable to conventionally used SCM in its capacity to support the proliferative and clonogenic potential of hUCPVCs and BMMSCs. We found that both hUCPVCs and BMMSCs cultured in StemMACS™ media proliferated as well as their SCM cultured counterparts. This is consistent with the findings of other groups that showed umbilical cord MSCs [43–48] and BMMSCs [49] exhibit similar or improved proliferation in serum- and xeno-free conditions over SCM. We also demonstrated that serum-free culture conferred greater clonogenic potential to hUCPVCs over SCM, which is consistent with previous studies on WJ MSCs in serum-free and serum-containing conditions [43–48]. Two notable exceptions to our findings and those of previous studies come from Wang et al. [50] and Chen et al. [51], wherein the proliferative capacity of umbilical cord MSCs was greater in SCM. Unlike what was observed for hUCPVCs, we did not find that the colony forming capacity of BMMSCs differed between StemMACS™ and SCM. The measured CFU-F frequency of BMMSCs in SCM was similar to that reported by Bhat et al., although they reported a significant decrease in clonogenicity in StemMACS™, when compared to SCM [49]. Nevertheless, our findings largely affirm trends reported in the literature, which overwhelmingly favour serum- and xeno-free culture for both umbilical cord MSCs and BMMSCs.

A direct comparison of the two cell populations in each culture condition showed that hUCPVCs proliferated more than their BMMSC counterparts, as evidenced by significantly higher cell counts and shorter population doubling times. Moreover, hUCPVCs had greater clonogenic potential than BMMSCs in StemMACS™. The superior proliferative and colony forming capacity of hUCPVCs over BMMSCs can be a result of different factors. First, BMMSCs experienced contact-inhibited growth after a confluent monolayer was produced, whereas hUCPVCs continued to grow in multiple layers (data not shown). This phenomenon was previously reported by Baksh et al. in SCM [19] and reinforced by a recent report from Bhat et al. that found cumulative population doublings decreased as a function of higher cell-seeding density of BMMSCs in both SCM and StemMACS™ [49]. Secondly, MSCs derived from adult tissue sources reach replicative senescence at lower passages than MSCs harvested from fetal or neonatal sources [52]. All experiments in this study were conducted with neonatal hUCPVCs at passage 3 and adult BMMSCs at passage 4 or 5. hUCPVCs have been reported to have a consistent CFU-F frequency over 10 passages in SCM [22]. Furthermore, studies of WJ MSCs revealed that a negligible proportion of senescent cells could be detected in serum and xeno-free media, whereas nearly 30% of WJ MSCs in SCM were senescent [43]. Previous findings suggest that replicative senescence of umbilical cord MSCs is either not detectable or delayed to late passages (> 10) [21, 50], whereas adult BMMSCs can undergo senescence as early as passage 7 [52].

In this study we found an absence of osteogenic and adipogenic differentiation potential among all hUCPVC donors. Previous studies on hUCPVCs [19], umbilical cord MSCs [21, 43–47, 50] and BMMSCs [46] have demonstrated that osteogenic and adipogenic differentiation potential of cells are retained regardless of whether the cells are isolated in SCM or serum- and xeno-free culture media. However, some groups have shown that compared to MSCs sourced from adult tissues, the adipogenic differentiation potential of neonatal hUCPVCs and WJ MSCs is significantly dampened as a result of delayed and reduced activation of the PPAR$\gamma$ transcription factor cascade [53], which is deemed essential to adipogenic differentiation. In the context of HVTE, the lack of osteogenic and adipogenic differentiation potential of hUCPVCs is favourable given that these lineages are associated with native valve pathology [54]. It should, however, be noted that the absence of osteogenic and adipogenic differentiation of hUCPVCs in vitro cannot predict how the cells will behave in situ.

The second objective of this study was to evaluate the capacity of StemMACS™ cultured hUCPVCs and BMMSCs to synthesize ECM proteins in vitro. The ECM proteins of the native PV - collagen, s-GAG, and elastin - are critical to its structure and function. Thus, in vitro HVTE strategies rely on MSCs to synthesize and organize the three constituent proteins of the ECM. We found that hUCPVCs deposited significantly more total collagen, s-GAG, and elastin than BMMSCs when cultured in StemMACS™. Cultures are often supplemented with AA to increase cell proliferation and collagen, s-GAG, and elastin deposition in a dose-dependent manner [55]. Here, we found no effect of AA supplementation of StemMACS™ on total DNA or ECM content, in either hUCPVCs or BMMSCs. It is likely that the proprietary StemMACS™ formulation itself contains AA and the cell response is saturated, rendering further supplementation with AA either inconsequential or pessimal to ECM synthesis. We can only speculate on this issue as the use of a commercial media with a proprietary formulation does not allow us to attribute the observed benefits to a single or specific combination of components. The greater total ECM synthesis capacity of hUCPVCs likely stems from its superior proliferative capacity over BMMSCs, as DNA-normalized collagen and s-GAG content, though greater than that of BMMSCs, was not found to be significantly different, whereas, the total cellularity and ECM protein content of hUCPVC cultures was significantly greater than BMMSCs. While the results of our studies favour the use of hUCPVCs over BMMSCs for in vitro HVTE, future works should also include hUCPVCs sourced from female donors to account for any sex-dependent differences in cell behaviour.

In a further assessment of their candidacy for in vitro HVTE, we cultured hUCPVCs on electrospun nanofibrous PCNU scaffolds with aligned fibres in StemMACS™. hUCPVCs retained their capacity to synthesize collagen, s-GAG, and elastin on nanofibrous PCNU. The DNA-normalized collagen, expressed as hydroxyproline, and s-GAG deposition reported here exceeded the maximum values reported previously for umbilical cord MSCs cultured on a synthetic scaffold to produce a living patch [24] and valved conduit [25]. The superior synthetic capacity of hUCPVCs in StemMACS™ under static conditions is notable, as these previous studies required SCM and mechanical conditioning of the engineered tissues in bioreactors to maximize ECM synthesis [24, 25]. Also favourable was that hUCPVCs adjacent to the scaffold aligned with the PCNU fibres. Aligned fibrous scaffolds are desirable for HVTE as they confer anisotropic tensile properties analogous to the native PV [56] and can direct cell and ECM alignment with the principal direction of the fibres [57] in order to mimic the orientation of collagen and elastin fibres in the native valve [58]. The off-axis alignment of cells in tissue layers away from the scaffold was ostensibly because of loss of contact guidance from the scaffold. This could be mitigated in the future by increasing the porosity of the scaffold to facilitate greater tissue infiltration amongst the fibres [59, 60] or by mechanical conditioning in a strain bioreactor [61], a strategy that has been shown to enhance cell and ECM alignment [62].

## Conclusion

Serum- and xeno-free StemMACS™ medium supported culture of both hUCPVCs and BMMSCs, but hUCPVCs demonstrated superior proliferative, clonogenic, and ECM synthesizing capacity in vitro. Similarly, hUCPVCs cultured in StemMACS™ media on electrospun PCNU valve tissue engineering scaffolds deposited tissue containing collagen, s-GAG and elastin on the scaffold surface, where they aligned with the scaffold fibres. Together, these findings support hUCPVCs as a promising autologous cell source for in vitro HVTE to address the clinical need in pediatric patients with CHD.

## Supplementary Information

**Additional file 1.** Supplemental Materials and Methods.

## Data Availability

The data that support the findings of this study are available from the corresponding author upon reasonable request.

## Abbreviations

AA: Ascorbic acid
ADO: Anionic dihydroxyl oligomer
ANOVA: Analysis of variance
BMMSC: Bone marrow-derived MSC
CFU-F: Colony forming unit-fibroblasts
CHD: Congenital heart defect
ECM: Extracellular matrix
GMP: Good manufacturing practices
hUCPVC: Human umbilical cord perivascular cell
HVTE: Heart valve tissue engineering
ISCT: International Society for Cell and Gene Therapy
MSC: Mesenchymal stromal cell
OCT: Optimal cutting temperature
PBS: Phosphate buffered saline
PCNU: Polycarbonate polyurethane
SCM: Serum-containing media
s-GAG: Sulfated glycosaminoglycan
TEHV: Tissue engineered heart valve
SEM: Standard error of the mean
