# Supplementary material for: Serum- and xeno-free culture of human umbilical cord perivascular cells for pediatric heart valve tissue engineering
Source: Stem Cell Res Ther. 2023 Apr 19;14:96. doi: 10.1186/s13287-023-03318-3 (PMC10116794; doi:10.1186/s13287-023-03318-3)
Supplement: Supplementary file 1 — Additional file 1. Supplemental Materials and Methods. [file 13287_2023_3318_MOESM1_ESM.docx]

**SUPPLEMENTAL INFORMATION**

**Serum- and xeno-free culture of human umbilical cord perivascular cells for pediatric heart valve tissue engineering**

Shouka Parvin Nejad^1,2^, Monica Lecce^1,2^, Bahram Mirani^1,2,3^, Nataly Machado Siqueira^1,2^, Zahra Mirzaei^1,3^, J. Paul Santerre^1,2,4^, John E. Davies^2,4,5^, Craig A. Simmons^1,2,3^

^1^Translational Biology and Engineering Program, The Ted Rogers Centre for Heart Research, Toronto, Canada.

^2^Institute of Biomedical Engineering, University of Toronto, Toronto, Canada

^3^Department of Mechanical and Industrial Engineering, University of Toronto, Toronto, Canada

^4^Faculty of Dentistry, University of Toronto, Toronto, Canada

^5^Tissue Regeneration Therapeutics, Toronto, Canada

**Correspondence to:** Craig Simmons ([c.simmons@utoronto.ca](mailto:c.simmons@utoronto.ca)) and Shouka Parvin Nejad ([shouka.parvinnejad@mail.utoronto.ca](mailto:shouka.parvinnejad@mail.utoronto.ca))

**Supplemental Materials and Methods**

***Papain and Oxalic Acid Digestion***

*hUCPVCs and BMMSCs on TCPS:* For papain digestion, cells were washed with PBS before the addition of 1 mL 40 *µ*g/mL papain (Sigma, cat. #P3125) solution in buffer (35 mM ammonium acetate, 1 mM ethylenediaminetetraacetic acid disodium salt dihydrate, 2mM dithiothreitol). Cells and deposited ECM were detached from wells using a cell scraper. Harvested samples in papain solution were collected in safe-lock Eppendorf tubes and incubated on a heat-block at 65$℃$ for 72 hours. Papain lysates were stored at -20$℃$ for further analysis. For each donor and media condition 8 technical replicates were harvested by papain digestion.

For digestion in oxalic acid, cells were washed twice with PBS, trypsinized, and centrifuged (3000 RPM, 10 minutes) according to the vendor protocol in the Fastin™ Elastin Assay Kit by Biocolor. The supernatant was aspirated and tissue was resuspended in 0.4 mL 0.25 M oxalic acid in safe-lock Eppendorf tubes. Samples were incubated on a heat-block at 100$℃$ for 1 hour and subsequently centrifuged (10,000 x g, 4$℃$, 10 minutes). The supernatant (oxalic acid lysate) was collected and stored at -20$℃$ for further analysis. For each donor and media condition 8 technical replicates were harvested by oxalic acid digestion.

*hUCPVCs on PCNU:* For papain digestion, constructs (N = 3) were washed with PBS, transferred to Eppendorf tubes with 1 mL 40 *µ*g/mL papain and incubated on a heat-block at 65$℃$ for 72 hours. Papain lysates were stored at -20$℃$ for further analysis. For digestion in oxalic acid, constructs (N = 3) were washed twice with PBS, transferred to Eppendorf tubes with 750 $\mu$L 0.4M oxalic acid, and incubated on a heat-block at 100$℃$ for 1 hour. Samples were centrifuged (10,000 x g, 4$℃$, 10 minutes) and the supernatant was stored at -20$℃$ for further analysis.

***DNA Quantification***

Papain digested samples were thawed on ice and 50 $\mu$L of lysate was aliquoted into a black wall, clear bottom 96-well plate in triplicate. Samples were mixed with 200 $\mu$L 0.1 $\mu$g/mL Hoechst 33258 dye (Life Technologies, cat. #H3569). The well plate was then transferred on ice to the plate reader and sample fluorescence was measured (excitation at 350 nm and emission at 450 nm). The net DNA content of the samples was quantified against calf thymus DNA (Sigma, cat. #D3664) standards of known concentrations. For each donor and media condition, the DNA content of 3 technical replicates was quantified.

***Sulphated Glycosaminoglycan Quantification***

Papain digested samples were thawed on ice and 20 $\mu$L of lysate was aliquoted into a 96-well plate in triplicate. Samples were mixed with 200 $\mu$L 0.016% dimethylmethylene blue dye (Sigma, cat. #341088) solution. The well plate was transferred to the plate reader and absorbance was measured at 525 nm. Sulfated glycosaminoglycan content was quantified against chondroitin sulfate standards (Sigma, cat. #C9819) of known concentrations. For each donor and media condition, the sulfated glycosaminoglycan content of 3 technical replicates was quantified.

***Hydroxyproline Quantification***

Papain digested samples were thawed on ice and 100 $\mu$L of lysate was mixed with 100 $\mu$L 6.0 N HCl in heat-resistant glass culture tubes with screw caps. The mixture was heated at 110$℃$ on a heat-block for 18 hours. The hydroxylates were taken off the heat-block and neutralized with 100 $\mu$L 5.7 N NaOH. 100 *µ*L of neutralized samples were aliquoted into a 96-well plate in triplicate. Aliquots were subsequently mixed with 50 $\mu$L 0.05 N chloramine-T (Sigma, cat. #857319) (20 minutes, room temperature), followed by 50 $\mu$L 3.15 N perchloric acid (5 minutes, room temperature) and finally 50 $\mu$L Ehrlich’s reagent (Sigma, cat. #39070) (20 minutes, 60$℃$). The well plate was cooled on ice and transferred to the plate reader. Absorbance was measured at 560 nm. Hydroxyproline content was measured against L-hydroxyproline (Sigma, cat. #H54409) standard solutions of known concentration. For each donor and media condition, the hydroxyproline content of 3 technical replicates was quantified.

***Insoluble*** $\boldsymbol{\alpha}$***-Elastin Quantification***

The Fastin™ Elastin Assay Kit by Biocolor (Accurate Chemical and Scientific Corporation, cat. # CLRF2000) was used to quantify insoluble $\alpha$-elastin content of OA lysates following the manufacturer’s protocol. For each donor and media condition, the $\alpha$-elastin content of 3 technical replicates was quantified.

***F-actin and Nuclei Staining***

Cell-seeded constructs were washed with PBS (with calcium and magnesium) and fixed with 10% neutral buffered formalin (NBF) (Sigma, cat. #HT501128) for 10 minutes at room temperature. Constructs were twice washed with PBS and stored at 4$℃$ until staining. hUCPVCs were permeabilized with 0.1% Triton X-100 (Sigma, cat. #T8787) in PBS for 10 minutes. Constructs were twice washed with PBS before blocking with 2% bovine serum albumin (Sigma, cat. #A9647) solution for 20 minutes at 37$℃$. Samples were washed with PBS and incubated with a 1:100 dilution of FITC-labelled phalloidin (Sigma, cat. #P5282) F-actin stain in 3% bovine serum albumin for 1 hour at room temperature. Samples were then washed with PBS and stained with a 1:500 dilution of Hoechst 33342 (Sigma, cat. #B2261) nuclear stain in PBS for 10 minutes at room temperature. Constructs were washed with PBS and stored in mounting media at 4$℃$ before imaging by confocal microscopy.
